# Enhancing Tip Detection by Pre-Training with Synthetic Data for Ultrasound-Guided Intervention

**DOI:** 10.3390/diagnostics15151926

**Published:** 2025-07-31

**Authors:** Ruixin Wang, Jinghang Wang, Wei Zhao, Xiaohui Liu, Guoping Tan, Jun Liu, Zhiyuan Wang

**Affiliations:** 1 Department of Radiology, The Second Xiangya Hospital, Central South University, Changsha 410011, China; rxwang@csu.edu.cn (R.W.); sighcat419@163.com (J.W.); wei.zhao@csu.edu.cn (W.Z.); junliu123@csu.edu.cn (J.L.); 2 The First People’s Hospital of Kunshan, Affiliated Kunshan Hospital of Jiangsu University, Suzhou 215300, China; lxhmedicine123@163.com; 3 College of Computer and Software, Hohai University, Nanjing 211100, China; gptan@hhu.edu.cn; 4 Department of Ultrasound, The Affiliated Cancer Hospital of Xiangya School of Medicine, Central South University, Changsha 410013, China

**Keywords:** needle tip detection, ultrasound-guided interventions, data synthesis, generative deep learning

## Abstract

**Objectives**: Automatic tip localization is critical in ultrasound (US)-guided interventions. Although deep learning (DL) has been widely used for precise tip detection, existing methods are limited by the availability of real puncture data and expert annotations. **Methods**: To address these challenges, we propose a novel method that uses synthetic US puncture data to pre-train DL-based tip detectors, improving their generalization. Synthetic data are generated by fusing clinical US images of healthy controls with tips created using generative DL models. To ensure clinical diversity, we constructed a dataset from scans of 20 volunteers, covering 20 organs or anatomical regions, obtained with six different US machines and performed by three physicians with varying expertise levels. Tip diversity is introduced by generating a wide range of synthetic tips using a denoising probabilistic diffusion model (DDPM). This method synthesizes a large volume of diverse US puncture data, which are used to pre-train tip detectors, followed by subsequently training with real puncture data. **Results**: Our method outperforms MSCOCO pre-training on a clinical puncture dataset, achieving a 1.27–7.19% improvement in AP_0.1:0.5_ with varying numbers of real samples. State-of-the-art detectors also show performance gains of 1.14–1.76% when applying the proposed method. **Conclusions:** The experimental results demonstrate that our method enhances the generalization of tip detectors without relying on expert annotations or large amounts of real data, offering significant potential for more accurate visual guidance during US-guided interventions and broader clinical applications.

## 1. Introduction

Ultrasound (US)-guided needle puncture is a versatile and commonly used technique in various interventional procedures, particularly for minimally invasive surgeries [1,2,3]. It allows precise targeting of internal structures with minimal trauma, making it essential for applications such as tissue biopsies, catheter placements, fluid drainage, and tumor ablation treatments [4,5,6,7]. As inadvertent puncture could lead to complications like vascular bleeding and organ damage, automatic needle tip localization in the US images is of relevance to the interventionalist, particularly the novice with insufficient experience. Using automatic tip localization, visual indicators for the needle tip can be directly overlaid on US images, assisting the interventionalist in performing free-hand needle punctures. This enhances visual guidance by allowing the interventionalist to hit the target with increased accuracy and ease.

To achieve automatic and precise tip localization in the US images, deep learning (DL)-based image processing techniques have been widely applied by researchers. Mwikirize et al. [8] utilize a DL-based object detection method, i.e., Fast R-CNN [9], to detect bounding boxes containing the needle shaft in US images as regions of interest (ROI). They then segment the needle shaft within the ROI and fitted the needle insertion direction. Finally, the tip position is obtained based on pixel intensity analysis. Chen et al. [10] designed the WNet segmentation network to segment the needle shaft first. Based on the segmented shaft position, the needle insertion direction is then fitted using the least squares method, and the deepest point on the segmented needle shaft is taken as the tip position. These methods reported minimal tip localization errors on puncture data from ex-vivo beef and pork samples conditioned on precise shaft localization and segmentation before tip localization. However, the shaft information is not always reliable: For out-of-plane punctures, visible needle shafts are not available and even for in-plane punctures, shafts could also be absent in deep positions from time to time due to the deviation from the thin beam plane. To address this issue, researchers localize tips directly without prior shaft information by utilizing the motion information contained in the dynamic US image stream [11,12,13,14]. In our previous study [15], an advanced tip detection framework called TipDet that uses long-and-short term spatiotemporal information to identify tips was proposed. TipDet achieved state-of-the-art tip detection performance on clinical data from multiple human organ punctures. However, when applied in real life for clinical application, TipDet could encounter degraded performance, particularly declined generalization regarding the limited clinical data (from 38 patients) it is developed with, which requires more labeled clinical puncture data to further optimize the model. Unfortunately, rich and diverse clinical puncture data with expert annotation are hard to acquire for the following reasons:

- Clinical US images have the features of equipment diversity, physician diversity, patient diversity, and tip diversity. Wherein the equipment diversity means that US puncture images are acquired from various grades of US machines from various manufacturers, leading to all kinds of image styles and quality. Physician diversity means US puncture images could have great diversity from scanners with various levels of scanning expertise and experience. Patient diversity denotes that US puncture images of various patients could be also different even for the same organ due to individual differences. Finally, tip diversity refers to the variation in echo characteristics of the tip, which changes with factors such as needle angle, tip material, ultrasound frequency, and the properties of surrounding tissues.
- Expert annotations for tips are quite expensive. To train TipDet and other DL-based tip detectors, tip bounding box annotation for thousands of images is necessary. Moreover, the more and the higher-quality annotations are acquired, the better tip detection performance is expected. However, the labeling process is monotonous, time-consuming, and exhausting, and the cost of hiring highly skilled interventionalists is prohibitive.

Recently, researchers have explored synthetic data generation, particularly diffusion-based models, to overcome data scarcity in medical image analysis. Khosravi et al. employed denoising diffusion probabilistic models (DDPMs) to generate synthetic chest X-ray images for pathology classification [16]. Wang et al. applied a latent stable diffusion model to generate multi-modality (OCT, Chest CT, Chest X-ray, and Fundus) medical images for diagnosis, report generation, and self-supervised learning [17]. Xu et al. developed CoLDiT, a conditional latent diffusion model to generate US images of breast lesions across various Breast Imaging Reporting and Data System (BI-RADS) categories [18]. Although synthetic data generation has been extensively studied for various tasks, to the best of our knowledge, there is no prior work specifically using synthetic data for US-guided needle tip detection.

To address the data acquisition challenges and provide more US puncture data for training DL-based tip detectors, thereby enhancing their robustness in complex clinical scenarios, we propose a data synthesis method that can generate a large volume of US puncture images with significant clinical diversity, without the need for expert labeling. In the proposed method, we first collected a large dataset of clinical US images (without tips) scanned by multiple physicians with varying levels of expertise, using a range of US machines. Moreover, these data were scanned from multiple volunteers with different individual characteristics. Thus, the collected data can provide all kinds of image backgrounds and ensure a certain level of clinical diversity. Second, we generate massive new tips through generative DL based on existing real tip annotations. In so doing, we can increase the tip variety beyond the original ones. Third, we fuse the collected clinical US data with the newly generated tips and acquire a lot of synthetic US puncture images. These synthetic US puncture images thus exhibit great clinical diversity regarding either the tip or the image background. More importantly, annotations of tip bounding boxes can be automatically generated without expert labeling. Finally, with the synthetic US puncture data, we pre-train tip detectors before training them with the limited real data. With the proposed method, we can enhance the performance, particularly the generalization of existing tip detectors in complex clinical environments without extra expert-labeled real data, so as to further provide convenience for interventionalists and enhance the usability of US-guided needle puncture. The key contributions of this study are threefold:

- We propose a data synthesis method capable of generating large volumes of US puncture images with substantial clinical diversity, all without the need for expert labeling, thereby significantly reducing the data acquisition costs for training advanced tip detectors.
- Using the proposed method, we generated a large dataset of synthetic US puncture images. Through pretraining with this synthetic data, we further enhanced the performance of the current tip detector, particularly improving its generalization capability, resulting in a new SOTA tip detector, TipDet with synthetic data pre-training (TipDet-SDP).
- To facilitate the research of automatic tip localization for US-guided interventions, we have released part of our research data and tip generation model.

The rest of the article is arranged as follows: Section 2 describes the synthesis method for US puncture data; Section 3 evaluates the effectiveness of the proposed method with extensive experiments on clinical puncture data; Section 4 concludes this study; Section 5 introduce the limitations and our future work.

## 2. Materials and Methods

**Figure 1:**
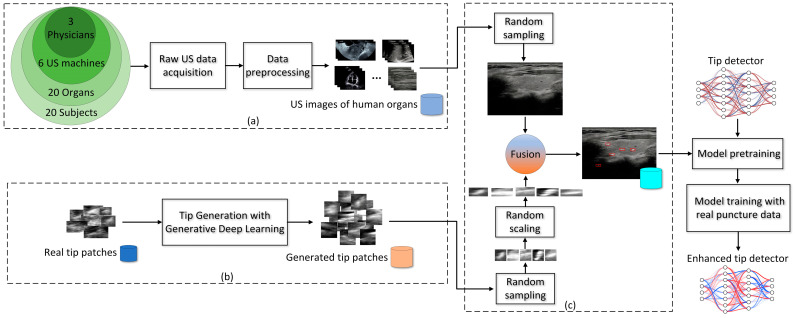
Diagram of the proposed method. (**a**) Clinical US image acquisition, (**b**) new tip generation, and (**c**) US puncture image synthesis.

As shown in Figure 1, to synthesize US puncture images, the proposed method consists of three stages: (a) Clinical US image acquisition, (b) new tip generation, and (c) US puncture image synthesis. Then, we enhance the current tip detectors using the synthesized images by pre-training the tip detector before training it subsequently with real puncture data. This pipeline also reflects the potential clinical workflow, where the trained model can be deployed as a lightweight module into US guidance systems for real-time tip detection during procedures.

### 2.1. Clinical US Image Acquisition

As mentioned above, US puncture images are characterized by great clinical diversity of equipment diversity, physician diversity, patient diversity, and tip diversity. Therefore, to ensure robust generalization of tip detectors across various clinical settings, the training data must encompass the clinical diversity. In this study, we introduce a method capable of synthesizing large volumes of US puncture data with substantial clinical diversity from two aspects: image background diversity and tip diversity. In this section, we first focus on providing diverse image backgrounds for the desired synthesized data.

**Figure 2:**
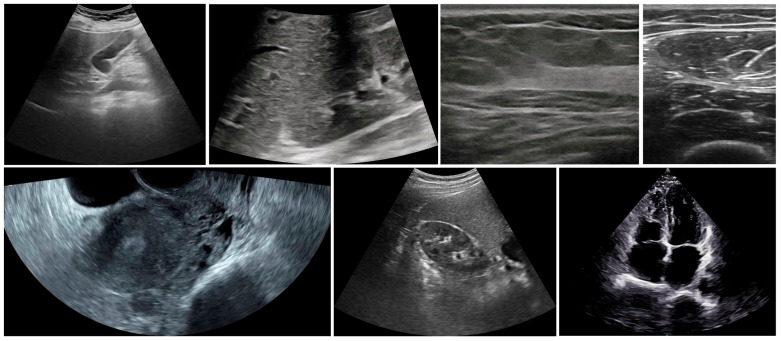
Examples of the CUID-HO-50k.

Figure 1a illustrates the construction of a large-scale clinical US image dataset of human organs or anatomical regions (referred to as CUID-HO in this study), which involves the following steps:

(1) Raw US video acquisition. To address patient diversity, we first recruited 20 volunteers with varying individual characteristics as scanned subjects. As shown in Table 1, three US physicians with 1, 5, and 10 years of clinical experience, respectively, were employed to scan 20 organs or anatomical regions of each subject using six different US machines. For each volunteer, each target organ or anatomical region was scanned by a randomly selected physician using a randomly chosen US machine. US videos were included if they were identified as healthy controls by the US physician. In total, 405 raw US videos were acquired. The involvement of multiple physicians, various US machines, and multiple subjects and organs contributes to the noticeable diversity of the CUID-HO dataset, ensuring the background diversity of the synthesized US puncture data. US scanning was performed in the First People’s Hospital of Kunshan, Jiangsu, China. Before US scanning, all volunteers were required to sign informed consent forms, and the procedure was approved by the hospital’s ethics committee.

(2) Data preprocessing. The 405 raw US videos were extracted frame by frame, yielding 245,585 raw US frames. To reduce temporal redundancy, one frame was sampled for every 5 frames, resulting in 49,117 frames. These images were then cropped, retaining only the central imaging region. Ultimately, we obtained the complete CUID-HO dataset, consisting of 49,117 grayscale frames, and specifically denoted it as CUID-HO-50k. Samples from the CUID-HO-50k are shown in Figure 2.

The construction of the dataset CUID-HO did not involve patients with specific diseases, allowing the entire dataset to be built within two weeks. To investigate the impact of dataset size on model performance, we created four additional subsets from CUID-HO-50k using four down-sampling intervals (20 frames, 10 frames, 5 frames, and 2 frames), designated as CUID-HO-2.5k, CUID-HO-5k, CUID-HO-10k, and CUID-HO-25k, respectively.

### 2.2. New Tip Generation with Generative DL

**Figure 3:**
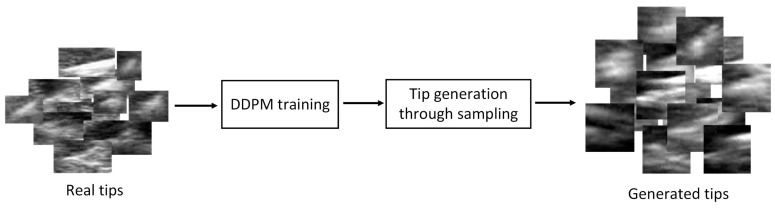
Tip generation procedures.

As illustrated in Figure 1b, in addition to constructing CUID-HO, we also generate new tips using generative DL to enhance tip diversity. In recent years, diffusion models have demonstrated remarkable capability in generating high-quality and diverse images with stable training and a solid theoretical foundation [19]. Thus, in this study, we utilize the classic DDPM [20] to generate new tips based on real tip data. As illustrated in Figure 3, tip generation consists of two steps: DDPM training and tip generation through sampling.

#### 2.2.1. DDPM Training

The DDPM generates images through a forward diffusion process and a reverse generation process. In the forward process, noise is progressively added to the image until the entire image becomes pure Gaussian noise. Specifically, this process can be expressed as:

(1) $$x_{t}=\sqrt{1-\beta_{t}}x_{t-1}+\sqrt{\beta_{t}}\epsilon_{t}$$

where *x_t_* denotes the image with noise added, subscript *t* is the time step, and *x*_0_ represents the original image. $\epsilon_{t}\sim 𝒩(0,I)$ is the noise image sampled from Gaussian noise. $\beta_{t}$ is a predefined positive value indicating the ratio of the added noise that gradually increases, i.e., $0<\beta_{1}<\ldots <\beta_{t}<\ldots <\beta_{T}<1$, where *T* (default 1000) is the total number of time steps. For simplicity, let $\alpha_{t}=1-\beta_{t}$ and ${\bar{\alpha }}_{t}=\prod_{i=1}^{t}\alpha_{i}$. In the reverse generation process, the DDPM attempts to recover the original image from pure Gaussian noise by progressively reducing the noise through the following equation:

(2) $$x_{t-1}∼𝒩(\frac{1}{\sqrt{\alpha_{t}}}(x_{t}-\frac{\beta_{t}}{\sqrt{1-{\bar{\alpha }}_{t}}}\epsilon_{\theta }(x_{t},t)),\frac{1-{\bar{\alpha }}_{t-1}}{1-{\bar{\alpha }}_{t}}\beta_{t}I)$$

where $\epsilon_{\theta }(x_{t},t)$ represents the denoising function capable of predicting the added noise at a given time step *t*. In the DDPM, a U-Net [21] structure is applied to act as the denoising function. The DDPM training process aims to optimize $\epsilon_{\theta }$ by minimizing the following mean squared error (MSE) so that the estimated noise is as close as possible to the added noise:

(3) $$L(\theta )=E_{t,x,\epsilon }[{\left\|\epsilon -\epsilon_{\theta }(x_{t},t)\right\|}^{2}]$$

For tip generation, we first acquire the tip image patches by cropping the regions of the tip bounding boxes from the real US puncture dataset SUID-HP that was constructed in our previous work [15]. Specifically, for each image in the dataset, we extract a rectangle patch centered on the annotated tip bounding box. To preserve contextual features, a padding of five pixels is added on all sides before cropping. The resulting patch is then resized to 64 × 64 pixels and all values are normalized to the range [−1, 1], consistent with the input requirement of the DDPM.

#### 2.2.2. Tip Generation Through Sampling

When the denoising function $\epsilon_{\theta }$ is learned through DDPM training, a new tip image patch can be generated through the sampling procedures as follows:

(1) Acquire an image patch $x_{T}$ through sampling from Gaussian noise: $x_{T}\sim 𝒩(0,I)$;
(2) Predict the noise added at time step *t* with $\epsilon_{\theta }$;
(3) Based on Equation (2), acquire the noise-added image $x_{t-1}$ at time step *t* through sampling:
  (4) $$x_{t-1}=\frac{1}{\sqrt{{\bar{\alpha }}_{t}}}(x_{t}-\frac{1-\alpha_{t}}{\sqrt{1-{\bar{\alpha }}_{t}}}\epsilon_{\theta }(x_{t},t))+\sigma_{t}z$$
  where the standard deviation $\sigma_{t}=\sqrt{\frac{1-{\bar{\alpha }}_{t-1}}{1-{\bar{\alpha }}_{t}}\beta_{t}}$ and z~𝒩(0,I);
(4) Repeatedly perform step (3) for *T* times to acquire $x_{0}$, i.e., a generated tip image patch.

**Figure 4:**
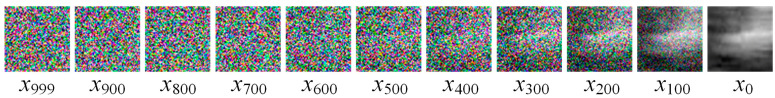
DDPM generation process of a tip image patch.

**Figure 5:**
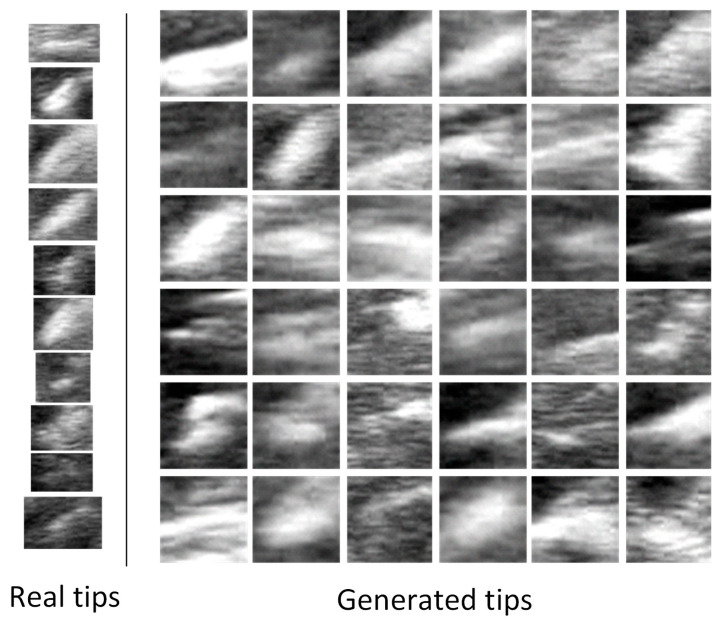
Generated tips versus real tips.

Figure 4 illustrates the intermediate generation results of a new tip image patch. It can be observed that as the denoising process progresses, the DDPM gradually generates image patches containing the tip. By repeating this process with various random Gaussian noise, a large number of image patches containing tips can be generated. In this study, we generated approximately 50,000 tip image patches, and these image patches form a dataset referred to as the Generated Dataset of Tips (GD-T). Figure 5 illustrates some generated tips with the DDPM. It can be seen that on the one hand, the generated tips are visually realistic compared with the real tips; on the other hand, the generated tips exhibit great diversity regarding tip morphology, size, brightness, and contrast.

#### 2.2.3. Puncture Image Synthesis

As illustrated in Figure 1c, when the clinical US image dataset CUID-HO and generated tip dataset GD-T are both prepared, we fuse them to synthesize a US puncture image. The image synthesizing procedures are as follows:

(1) Data sampling. Randomly sample a US image from CUID-HO, denoted as *I*. Simultaneously, randomly sample five generated tip image patches from GD-T. The aim of sampling five image patches rather than one is to increase the number of positive samples for the tip during detector training, alleviating the imbalance between positive and negative samples and reducing the learning difficulty for the detector.

(2) Random scaling. Each of the five sampled tip image patches is randomly scaled with the target width (*w_s_*) and height (*h_s_*) drawn from the following normal distributions:

(5) $$f(h_{s})=\frac{1}{6\sqrt{2\pi }}\exp\left(-\frac{1}{2}{\left(\frac{h_{s}-20}{6}\right)}^{2}\right)$$

(6) $$f(w_{s})=\frac{1}{10\sqrt{2\pi }}\exp\left(-\frac{1}{2}{\left(\frac{w_{s}-36}{10}\right)}^{2}\right)$$

where *h*_s_ corresponds to a distribution mean of 20 and a standard deviation of 6; *w_s_* corresponds to a distribution mean of 36 and a standard deviation of 10. The target height (*h*_s_) and width (*w_s_*) used for random scaling were derived from a statistical analysis of the tip bounding boxes in the SUID-HP dataset. Specifically, we computed the mean and standard deviation of the height and width across all annotated bounding boxes in the training set (15,517 samples). The average height and width were found to be approximately 20 pixels and 36 pixels, respectively, with corresponding standard deviations of 6 and 10 pixels. We chose this distribution-based sampling strategy to introduce realistic size variability into the synthetic tip patches while preserving consistency with real clinical data.

(3) Image fusion. Randomly select five positions in *I* using a uniform distribution and apply Poisson Editing [22] to fuse the five random scaled tip image patches at these positions. Poisson Editing is selected as it can adapt to the brightness and contrast conditions of the target image (i.e., the clinical US images) while preserving the details of the source image (i.e., the tip image patches). Finally, a clinical US image containing five generated tips can be synthesized.

**Figure 6:**
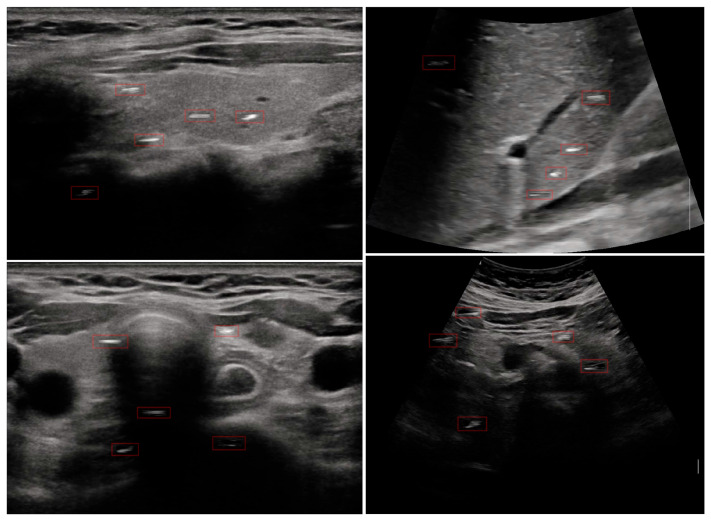
Synthetic US puncture images. Each contains five generated tips rRed bounding boxes).

Figure 6 shows four examples of the synthetic US images. It can be seen that the CUID-HO dataset provides diverse clinical images as background for the synthetic data, while the newly generated tips offer richer and more diverse targets. Thus, the synthetic data can address the equipment diversity, physician diversity, patient diversity and tip diversity of clinical US puncture images, enhancing the generalization of tip detectors in complex clinical environments. In this study, we synthesized multiple scales of tip datasets from corresponding CUID-HO datasets, denoted as CUID-HO-50k-T, CUID-HO-25k-T, CUID-HO-10k-T, CUID-HO-5k-T, and CUID-HO-2.5k-T, respectively.

#### 2.2.4. Utilization Methods of the Synthetic Data

**Figure 7:**
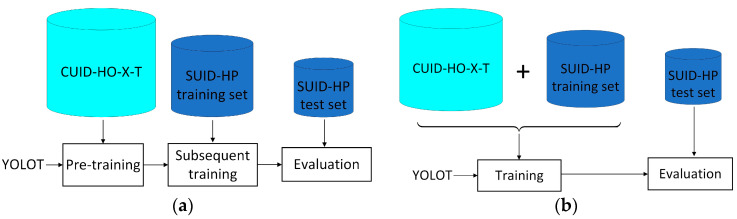
Utilization method of the synthetic data. (**a**) Pre-training (Setting P); (**b**) Direct training (Setting D).

As shown in Figure 7, we studied the effectiveness of the synthetic US puncture data under two settings:

(1) Pre-training (denoted as setting P hereafter). We pre-train the YOLOT detector on the synthetic dataset, then continue training the pre-trained model with SUID-HP training set. On SUID-HP test set, the final model is evaluated. YOLOT is the base detector used in TipDet, which results from removing the smallest scale of feature maps and the feature pyramid network (FPN) of YOLOX-Nano [23]. During pre-training, the synthetic dataset is divided into training and validation sets in a 9:1 ratio, with the validation set used to determine whether to apply early stopping during the pre-training process.

(2) Direct training (denoted as setting D hereafter). In this manner, we merge the synthetic dataset with SUID-HP training set and then train YOLOT using the merged dataset, followed by evaluation on SUID-HP test set.

## 3. Experimental Results and Discussions

### 3.1. Experimental Setup

#### 3.1.1. Real US Puncture Dataset

As mentioned above, for the real US puncture data, we use SUID-HP, a clinical human puncture dataset collected from the First People’s Hospital of Kunshan, Jiangsu, China. SUID-HP contains 21,282 grayscale images from punctures of 38 patients, with each image containing an expert-labeled tip bounding box. The images were acquired from multiple organs, including thyroid, abdomen, breast, lymph, and testis. The training set of SUID-HP includes 15,517 images, the validation set consists of 1570 images and the test set contains 4195 images. Please refer to [15] for more details about SUID-HP.

#### 3.1.2. Evaluation Criterion

Tip detection. As in [15], AP_0.1:0.5_ is also applied in this study to evaluate the tip detection performance, defined as follows:

(7) $${\mathrm{AP}}_{0.1:0.5}=\frac{1}{5}\sum_{i\in \{0.1,\ldots ,0.5\}}{\mathrm{AP}}_{i}$$

where AP*_i_* (*i*∈ [0.1, 0.2, 0.3, 0.4, 0.5]) is the 101-point interpolated average precision computed with the method in [24].

Tip localization. The localization performance of tip detectors is further evaluated using the root mean square error (RMSE) between the centers of the detected tip bounding boxes $\left({\tilde{x}}_{n},\;{\tilde{y}}_{n}\right)$ and the corresponding ground truth $\left(x_{n},\;y_{n}\right)$:

(8) $$\mathrm{RMSE}=\sqrt{\frac{1}{n_{b}}\sum_{n=1}^{n_{b}}\left[{\left({\tilde{x}}_{n}-x_{n}\right)}^{2}+{\left({\tilde{y}}_{n}-y_{n}\right)}^{2}\right]}$$

where *n*_b_ is the number of the detected tip bounding boxes.

#### 3.1.3. DDPM Training Setup

For the U-net model used to predict forward noise in the DDPM, we directly adopt the training settings of the original paper: total time steps *T* = 1000; linearly increasing *β_t_* with *β*_1_ = 10^−4^ and *β_T_* = 0.02; image input and generation resolution of 64 × 64, with pixel values linearly mapped to the range [−1, 1]; batch size of 32 and total 7500 training batches; learning rate of 10^−4^; AdamW optimizer (*β*_1_ = 0.9, *β*_2_ = 0.999).

#### 3.1.4. Tip Detector Training Setup

For both the pre-training and subsequent training phases in setting P, as well as the single training phase in setting D, the training settings described below are used. The difference is that in the pre-training phase of setting P and the single training phase of setting D, pre-trained weights from the MSCOCO dataset [24] pre-training are used to initialize the YOLOT; whereas in the subsequent training phase of setting P, model training continues based on the pre-trained model obtained in the pre-training phase.

Input transformation: The raw US images are first resized to 640 × 640 pixels, then expanded to three-channel images through channel replication, followed by random brightness perturbation that multiplies the raw pixel values with a random value sampled from the range of [0.9, 1.1] for data augmentation. Last, the z-score method is applied for data standardization.

DL training settings: The maximum number of training epochs was set to 100, with a batch size of 128; the Adam optimizer was used (*β*_1_ = 0.9, *β*_2_ = 0.999); the initial learning rate was set to 1 × 10^−3^ with a cosine learning rate strategy; the weight decay rate was set to 5 × 10^−4^; a warm-up training of three epochs was applied; an early stopping strategy with a patience value of 20 epochs was also implemented.

### 3.2. Effectiveness of the Synthetic Puncture Data

#### 3.2.1. Utilization Method Comparison

Tip detection performances of YOLOT under two utilization settings are shown in Table 2 and Table 3. First, in both settings, it can be observed that as the scale of the synthetic dataset increases, the detection performance of YOLOT on SUID-HP test set improves first and then declines. When using the CUID-HO-50k-T, the detection performance of YOLOT is the worst. Second, in setting P, the performance of YOLOT can exceed that of the MSCOCO pre-trained model: When pre-trained with CUID-HO-10k-T, YOLOT achieves an AP_0.1:0.5_ of 70.71%, surpassing MSCOCO pre-training by 0.9%; with CUID-HO-25k-T pre-training, YOLOT reaches an AP_0.1:0.5_ of 70.29%, exceeding MSCOCO pre-training by 0.48%. In comparison, when directly trained with the merged dataset under setting D, YOLOT is unable to achieve better tip detection performance than the MSCOCO pre-trained model.

**Figure 8:**
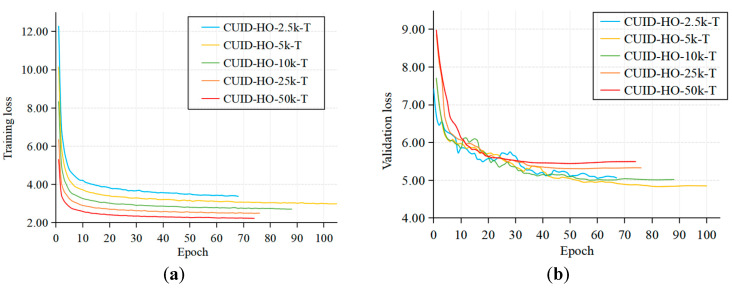
Loss curves during model training under setting D. (**a**) Training loss; (**b**) validation loss.

**Figure 9:**
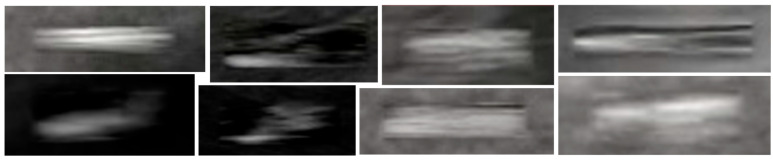
Tip areas in the synthetic images.

Figure 8 shows the loss curves for the training and validation sets during the training process under setting D. It can be observed that as the scale of the synthetic dataset in the merged dataset increases, the training loss curve gradually declines, while the validation loss curve first decreases and then increases. This phenomenon indicates that with more synthetic data used directly for training, the detector starts to overfit, reducing its generalization on the validation set. Moreover, the more synthetic data used, the more severe the overfitting. A possible reason for this could be the special patterns of fusion artifacts created at the boundaries of image patches by Poisson Editing. As shown in Figure 9, although Poisson Editing yields good overall image fusion results, particularly with smooth edge transitions, noticeable fusion artifacts emerge when there are significant texture differences between the tip image patches and the image background (as seen in the upper left image). When the scale of the synthetic dataset is relatively large, more fusion artifacts will be contained, leading the detector to focus on these specific embedding traces rather than the features of the tip itself. Conversely, a smaller synthetic dataset contains fewer fusion artifacts, resulting in slightly better detection performance. In contrast, under setting P, although the detector may also be misled by fusion artifacts during the synthetic dataset pre-training phase, the negative impact of these artifacts can be reduced since the synthetic dataset does not directly participate in the subsequent training on real puncture data. This allows the model to learn prior visual features about various clinical US images through pre-training with the synthetic dataset. Compared to using the MSCOCO pre-trained model, YOLOT shows higher detection accuracy on SUID-HP test set, indicating its better feature extraction and expression for clinical US puncture images, leading to improved generalization on unseen test sets. The experimental results demonstrate that the proposed method can further enhance the tip detection performance of existing detectors.

#### 3.2.2. Impact of the Number of Fused Tips

**Figure 10:**
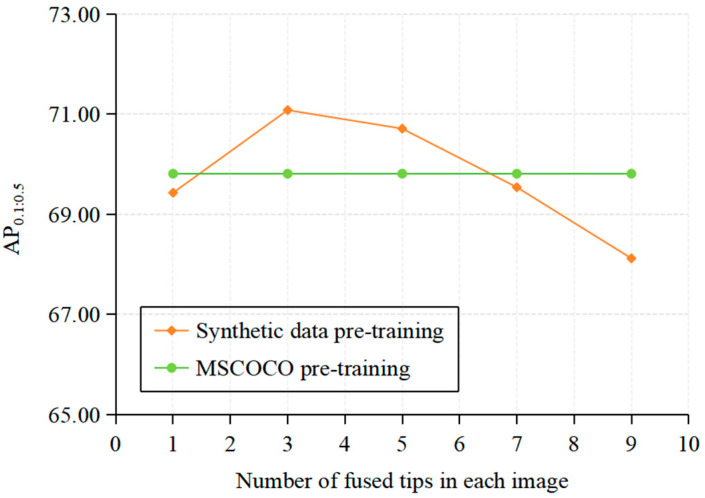
Impact of the number of tips fused per image on the effectiveness of the synthetic dataset under setting P.

The above experimental results are acquired when each synthetic image contains five generated tips. As analyzed earlier, image synthesis can produce fusion artifacts, thus the number of generated tips fused in the images affects the effectiveness of the synthetic dataset. Thus, in the pre-training phase under setting P, we further vary the number of fused tips in each image of the CUID-HO-10k-T to study the impact of tip quantity on the pre-training performance. As shown in Figure 10, as the number of fused tips per image increases, the pre-training performance first improves and then declines, consistent with the trends reflected in Table 2 and Table 3. When the number of fused tips is three, the synthetic data achieves the best performance (71.08%) with an improvement of 1.27% over MSCOCO pre-training (69.81%) in terms of AP_0.1:0.5_. Specifically, we denote the CUID-HO-10k-T corresponding to three fused tips per image as CUID-HO-10k-T (*n* = 3).

#### 3.2.3. Model Generalization

**Figure 11:**
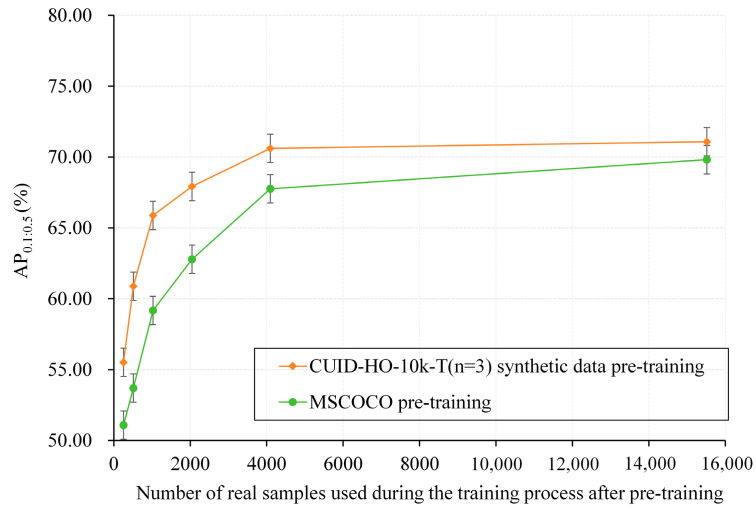
Performance comparison of the models with CUID-HO-10k-T pre-training and MSCOCO pre-training with various numbers of real training samples. The error bars indicate the standard deviation over three experimental runs with different random seeds.

To investigate the impact of synthetic data pre-training on model generalization, smaller sets of real training samples (256, 512, 1024, 2048, and 4096) were obtained from SUID-HP training set through equal interval sampling. Under setting P, YOLOT was further trained on these limited real training samples (15,517 samples) after CUID-HO-10k-T (*n* = 3) pre-training and MSCOCO pre-training. Then, the model’s generalization capability was evaluated based on YOLOT’s performance on the complete SUID-HP test set (4195 samples). As shown in Figure 11, first, with the same number of real training samples, the performance of the CUID-HO-10k-T (*n* = 3) pre-trained model is significantly better (1.27–7.19%) than that of the MSCOCO pre-trained model, indicating that the former has learned superior prior visual features regarding US images and tips, achieving better generalization. Second, compared to using the MSCOCO pre-trained model, the CUID-HO-10k-T (*n* = 3) pre-trained model demonstrates better performance with fewer real training samples. For example, using 256 real training samples, the synthetic data pre-trained model achieves an AP_0.1:0.5_ of 55.51% + 0.40%, while the MSCOCO pre-trained model only reaches 51.08% + 0.45%. Even with 512 training samples, the model only achieves 53.69% + 0.39%, and the performance differences are observed with other sample sizes. These results further indicate that pre-training with CUID-HO-10k-T (*n* = 3) can enhance model generalization.

**Figure 12:**
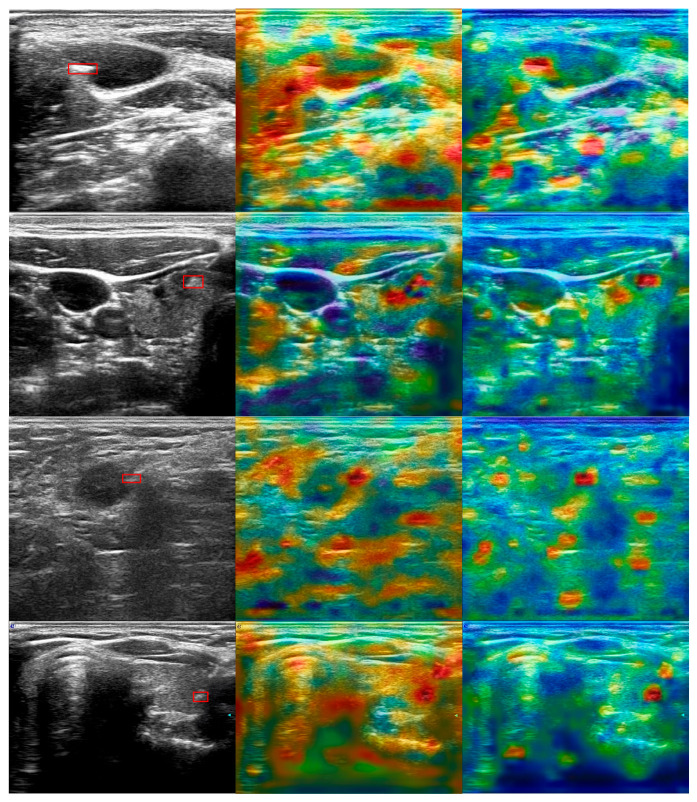
Activation map visualization. **Left**: Raw US images. **Middle**: Activation maps of YOLOT with MSCOCO pre-training. **Right**: Activation maps of YOLOT with CUID-HO-10k-T (*n* = 3) pre-training. Red bounding boxes: Ground truths for the tips.

In Figure 12, we illustrate the activation maps of YOLOT with MSCOCO pre-training and CUID-HO-10k-T (*n* = 3) pre-training under setting P. After pre-training, all real samples of the SUID-HP dataset are further used to train the detector. The activation maps are generated by first computing the confidence score (object confidence), the class score (the likelihood of the tip class), and the combined score (the product of the formers) of the 40 × 40 feature scale. These scores are then normalized to the [0, 1] range to ensure consistency across scales. Afterward, the normalized scores are averaged and transformed into a heatmap. Last, resize the heatmap to match the original image dimensions and overlay it onto the raw image. As illustrated in the activation maps, with CUID-HO-10k-T (*n* = 3) pre-training, first, fewer regions are highly activated (red areas) besides the tips, indicating that it is easier for the model to differentiate tips from interferences compared with MSCOCO pre-training, which would lead to fewer false positives. Second, regarding tip activation, the activation regions are more focused on the tip, which would result in more precise tip bounding boxes. The activation map results further indicate the superiority of CUID-HO-10k-T (*n* = 3) pre-training compared with MSCOCO pre-training.

The above experimental results indicate the effectiveness of the proposed method capable of enhancing tip detection without extra real puncture data of humans and expert annotations, thus showing substantial application potential for clinical practice.

#### 3.2.4. Ablation Study

To evaluate the effectiveness of clinical US data from healthy controls (CUID-HO) and DDPM-generated tip patches in the proposed method, we conducted the following two data synthesis approaches for tip detector pre-training: (1) Fuse images from CUID-HO-10k with real tip patches cropped from images in SUID-HP. (2) Fuse real puncture images from SUID-HP with DDPM-generated tip patches. For the two approaches, three tip patches (real or generated) were fused for each image following the procedures proposed in Section 2.2.3. The real tip patches were cropped applying the steps described in Section 2.2.1.

**Figure 13:**
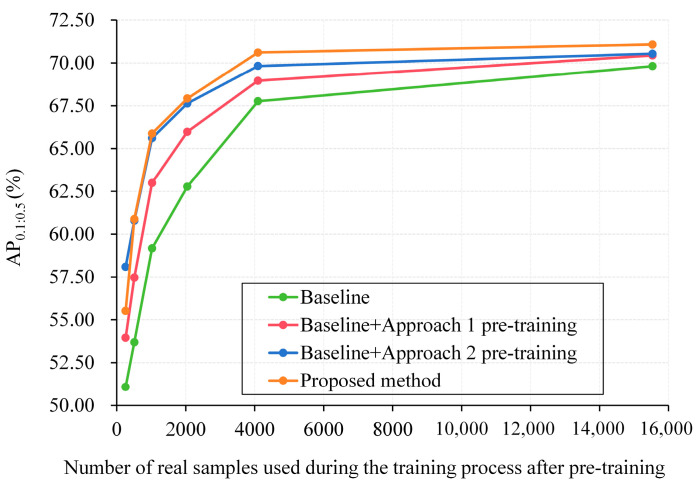
Effectiveness of clinical US data and DDPM-generated tip patches on tip detection. Baseline: MSCOCO initialization. Proposed method: CUID-HO-10k-T (*n* = 3) pre-training.

As illustrated in Figure 13, compared with the baseline of MSCOCO initialization (green line), both Approach 1 (red line) and Approach 2 (blue line) significantly improve tip detection performance, especially under low-data regimes (i.e., ≤2048 real samples). Notably, Approach 2, which utilizes DDPM-generated tip patches, consistently outperforms Approach 1 across all sample sizes, indicating the superiority of generative data over real patch fusion. Furthermore, our proposed method (orange line), which integrates both CUID-HO clinical data and DDPM-generated tip patches in a unified synthesis pipeline, achieves the highest AP_0.1:0.5_ scores under most training sample scales (except 256). This demonstrates that the complementary use of clinically diverse backgrounds and generatively diverse tip patterns contributes to more effective pre-training, leading to better generalization in downstream real-data application.

#### 3.2.5. Turing Test-Style Evaluation by US Physicians

To assess the perceptual realism of the DDPM-generated tips, we conducted a Turing test-style evaluation involving two senior US physicians (Physician 1: X.L. 13 years of expertise, Physician 2: Z.W. 20 years of expertise). In each test, US physicians were shown 100 image patches (50 real and 50 generated) that are randomly selected from the datasets of GD -T and SUID-HP, respectively. The selected patches were mixed and anonymized. The task was to independently judge whether each tip image patch was real or generated, based solely on its appearance. No contextual cues, anatomical labels, or source ratios were provided.

Table 4 illustrates the Turing-style test results. Each of the two physicians performed three independent rounds of evaluation. Each round used a different set of 100 samples. The values represent the percentage of generated tip patches misjudged as real, and real tips correctly identified as real. The relatively high fooling rates suggest strong perceptual realism of the generated tips, even to experienced clinicians.

#### 3.2.6. Tip Generation Efficiency and Deployment Feasibility

On the hardware platform of an NVIDIA RTX 3090 GPU and AMD 5950X CPU, under the setting of 1000 diffusion steps (*T* = 1000), generating a single tip patch (batch size = 1) takes approximately 18 s. For a batch generation (batch size = 256), the total time was 15 min and 43 s, yielding an average of 3.7 s per patch. Although the sampling time is relatively high, the synthetic data are generated offline prior to tip detector training, making it suitable for large-scale data augmentation. For faster tip generation, accelerated sampling strategies (e.g., DDIM [25] and Fast-DDPM [26]) and more powerful hardware can be applied.

Furthermore, the proposed method is fully software-based and compatible with existing DL-based US systems. Once the synthetic data are generated and the model is pre-trained, the final model can be deployed as a lightweight update without modifying US hardware or disrupting clinical workflows. This makes it highly suitable for real-world US-guided interventions, particularly in data-limited clinical environments. By improving tip visibility, our method can assist novice operators in achieving safer and more accurate puncture procedures.

#### 3.2.7. Enhancing Current Tip Detectors with Synthetic Pre-Training

To further verify the effectiveness of the proposed method, we applied the proposed method to SOTA video object detectors (as US imaging is dynamic), i.e., we pre-trained these detectors with CUID-HO-10k-T (*n* = 3) before training and evaluating them on SUID-HP. As illustrated in Table 5, all the SOTA detectors exhibit improvements when applying synthetic data pre-training (SDP). Particularly, the tip detection performance of TipDet is further enhanced by 1.76%, achieving an AP_0.1:0.5_ value over 80%, making TipDet-SDP the most advanced tip detector to date, to the best of our knowledge. As the dataset SUID-HP is constructed from clinical punctures, the enhancement of these detectors means their greater potential to be applied in real clinical practice. More robust and precise visual cues for tips can thus be provided to interventionalists, aiding them in performing US-guided puncture procedures with enhanced ease and safety.

**Table 5:** Performance of SOTA detectors with CUID-HO-10k-T (*n* = 3) pre-training.

Tip Detector	AP_0.1:0.5_ (%)	AP_0.2_ (%)	AP_0.5_ (%)	RMSE (Pixels)
MEGA-r50 [27]	70.07 ± 0.25	73.27 ± 0.27	61.93 ± 0.30	12.00 ± 6.95
**MEGA-r50-SDP**	**71.21** ± 0.22**↑1.14**	**74.35** ± 0.25	**62.94** ± 0.28	**11.22** ± 6.58
YOLOV-s [28]	68.67 ± 0.28	73.28 ± 0.32	57.09 ± 0.35	13.73 ± 6.98
**YOLOV-s-SDP**	**70.25** ± 0.26**↑1.58**	**75.38** ± 0.28	**59.46** ± 0.33	**11.89** ± 6.64
PTSEFormer-r101 [29]	77.74 ± 0.20	83.03 ± 0.22	64.61 ± 0.25	9.34 ± 5.40
**PTSEFormer-r101-SDP**	**78.96** ± 0.17**↑1.22**	**84.21** ± 0.24	**65.73** ± 0.23	**9.01** ± 5.54
TipDet [15]	78.72 ± 0.18	83.25 ± 0.20	66.51 ± 0.23	8.71 ± 4.25
**TipDet-SDP**	**80.48** ± 0.15**↑1.76**	**84.77** ± 0.18	**68.44** ± 0.21	**7.78** ± 4.10

Note: The mean and standard deviation are acquired by repeating the experiments three times with different random seeds. **↑1.14**: Improved by 1.14 %.

**Figure 14:**
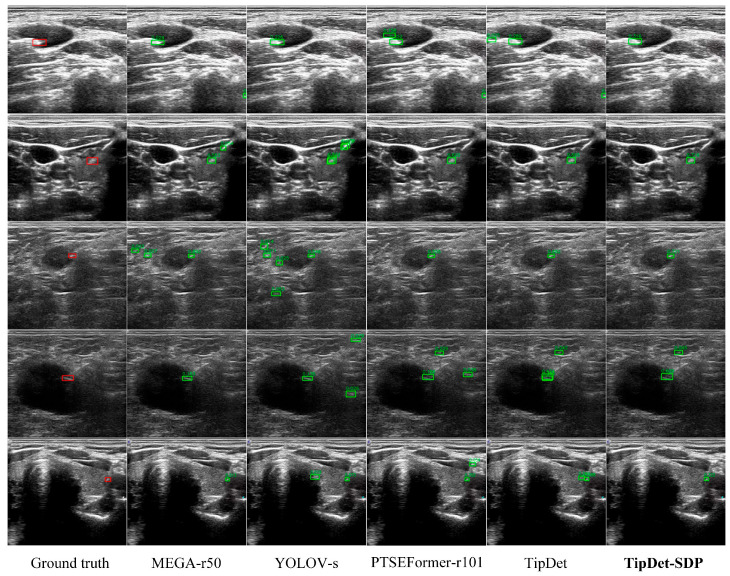
Detection examples of TipDet-SDP and SOTA VOD detectors. For the convenience of observation, the detection region is enlarged in the supplement material. Red bounding boxes: Ground truths for the tips. Green bounding boxes: Model predictions.

Figure 14 presents several examples of tip detection by TipDet-SDP and other SOTA detectors. Overall, TipDet-SDP outperforms current detectors across various organ punctures, showing a reduction in low-confidence false positives and an improvement in detection confidence for true positives. To further enhance model robustness and generalization, emerging techniques such as domain adaptation [30] may serve as a complementary strategy. In parallel, advances in ultra-high frequency ultrasound (UHF-US) also show potential for improving tip visibility in clinical settings [31].

## 4. Conclusions

In this study, to overcome the limited availability of real US puncture data and the high cost of expert annotations for training tip detectors, which further contribute to insufficient model generalization in complex clinical scenarios, we propose a method that first synthesizes a large volume of US puncture images (i.e., CUID-HO-X-T) and then pre-trains tip detectors with these synthetic data before training them subsequently on the limited real puncture data. The proposed method introduces no inference-time overhead on tip detection and can be integrated into real-time systems, as synthetic data are generated offline for pretraining only. The experimental results demonstrate that the proposed method can enhance the generalization of the tip detector, achieving more accurate tip detection on real puncture data. Additionally, the proposed method enables the development of a more advanced tip detector (i.e., TipDet-SDP). Thus, our study presents a novel solution to the challenges of US puncture data acquisition and expert labeling in the context of DL-based tip detection, so that we can provide more precise visual cues of tip localization for interventionalists, ultimately improving the ease and safety of US-guided interventional procedures.

## 5. Limitations and Future Work

First, as discussed in Section 3.2.1, we hypothesize that fusion artifacts in Poisson Editing result in the failure of the synthetic data utilization method of setting D. In the future, we aim to develop a more advanced image fusion technique tailored for US puncture data to mitigate these artifacts, further investigate the impact of these artifacts and explore the potential of higher-quality synthetic puncture data in the context of our proposed method. With improved synthetic data, we anticipate achieving more robust tip detection. Second, due to time and resource constraints, both the number of subjects and the number of US physicians involved in this study are limited. We are committing to construct a multi-center US dataset that involves more physicians and patients. A more diverse synthetic dataset is also expected. Lastly, the real dataset evaluation covers diverse anatomical areas but lacks confirmed pathological labels. In future work, we plan to incorporate pathological backgrounds and challenging anatomical regions for broader evaluation, and further perform system-level validation including prospective clinical studies and deployment efficiency analysis.

## Figures and Tables

**Table 1:** Property of the collected raw US videos.

Property	Number	Description
Raw videos	405	Mean video lasting time: 27 s, mean frame rate: 23, mean video width: 1108, mean video height: 785
Subject	20	Age range: 20–55, mean age: 37, male number: 10, female number: 10
Organs or anatomical region	20	Thyroid, carotid artery, heart, kidney, spleen, pancreas, liver, lung, gallbladder, breast, bladder, prostate (uterus), vertebral artery, femoral artery (vein), anterior tibial (posterior tibial) artery, popliteal artery (vein)
US physician	3	1 year, 5 years and 10 years of clinical practice
US machine	6	GE Vivid E95 (General Electric, Chicago, IL, USA), Philips IE33 (Philips, Amsterdam, The Netherlands), Samsung RS80 (Samsung, Suwon, Republic of Korea), Esaote Mylab Class C (Esaote, Genoa, Italy), Supersonic AixPlorer (Supersonic, Aix-en-Provence, France), and SonoStar UProbe C4PL (SonoStar, Guangzhou, China).

**Table 2:** Tip detection performance of YOLOT under setting P (%).

Pre-Training Dataset	AP_0.1:0.5_	AP_0.2_	AP_0.5_
None * (MSCOCO)	69.81	73.32	60.05
CUID-HO-2.5k-T	66.17	68.78	59.31
CUID-HO-5k-T	67.70	70.99	58.49
CUID-HO-10k-T	**70.71**	**73.51**	**62.09**
CUID-HO-25k-T	70.29	73.44	61.02
CUID-HO-50k-T	64.39	67.59	56.47

*: No synthetic data were used for pre-training; instead, the MSCOCO pre-trained model was directly trained on the SUID-HP training set.

**Table 3:** Tip detection performance of YOLOT under setting D (%).

Synthetic Dataset in the Merged Dataset	AP_0.1:0.5_	AP_0.2_	AP_0.5_
None * (MSCOCO)	**69.81**	**73.32**	**60.05**
CUID-HO-2.5k-T	67.63	71.05	58.51
CUID-HO-5k-T	68.55	71.95	58.79
CUID-HO-10k-T	66.73	70.26	56.13
CUID-HO-25k-T	62.28	65.14	54.13
CUID-HO-50k-T	61.44	64.09	54.24

*: No synthetic data were used; the MSCOCO pre-trained model was directly trained on the SUID-HP training set.

**Table 4:** Turing-style evaluation of synthetic tip realism by senior US physicians.

US Physician	Generated → Judged Real	Real → Judged Real
1	65.3% ± 13.20%	74.0% ± 9.93%
2	52.7% ± 11.81%	64.7% ± 8.22%
Average	59.0% ± 12.51%	69.4% ± 9.08%

## Data Availability

The codes and data are available at https://github.com/ResonWang/US-Puncture-Data-Synthesis- (accessed on 1 June 2025).

## Abbreviations

The following abbreviations are used in this manuscript:

SDP	Synthetic data pre-training
CUID	Clinical US image dataset of human organs or anatomical regions
DDPM	Denoising probabilistic diffusion model
GD-T	Generated dataset of tips

## References

[1] Müller T., Braden B. (2024). Ultrasound-guided interventions in the biliary system. Diagnostics.

[2] Huang Y.C., Lu Y.H., Ting W.Y. (2025). Ultrasound-guided vs. Non-ultrasound-guided femoral artery puncture techniques: A comprehensive systematic review and meta-analysis. Ultrasound J..

[3] Qafesha R.M., Kashbour M., Amro S., Hindawi M.D., Elbadry M., Ghalwash A.A., Alnatsheh Z., Abdelaziz M.A.Y., Eldeeb H., Shiha A.R. (2025). Ultrasound-guided thermal ablation versus thyroidectomy in the treatment of benign thyroid nodules: Systematic review and meta analysis. J. Ultrasound Med..

[4] Grasso F., Capasso A., Pacella D., Borgia F., Salomè S., Capasso L., Raimondi F. (2022). Ultrasound guided catheter tip location in neonates: A prospective cohort study. J. Pediatr..

[5] Gomaa S.M.A., Farouk M.H., Ali A.M. (2023). Ultrasound Guided Drainage and Aspiration of Intra-Abdominal Fluid Collections. Benha J. Appl. Sci..

[6] Che H., Qin J., Chen Y., Ji Z., Yan Y., Yang J., Wang Q., Liang C., Wu J. (2024). Improving Needle Tip Tracking and Detection in Ultrasound-Based Navigation System Using Deep Learning-Enabled Approach. IEEE J. Biomed. Heal. Inform..

[7] Bernardi S., Palermo A., Grasso R.F., Fabris B., Stacul F., Cesareo R. (2021). Current status and challenges of US-guided radiofrequency ablation of thyroid nodules in the long term: A systematic review. Cancers.

[8] Mwikirize C., Nosher J.L., Hacihaliloglu I. (2018). Convolution neural networks for real-time needle detection and localization in 2D ultrasound. Int. J. Comput. Assist. Radiol. Surg..

[9] Ren S., He K., Girshick R., Sun J. (2016). Faster R-CNN: Towards real-time object detection with region proposal networks. IEEE Trans. Pattern Anal. Mach. Intell..

[10] Chen S., Lin Y., Li Z., Wang F., Cao Q. (2022). Automatic and accurate needle detection in 2D ultrasound during robot-assisted needle insertion process. Int. J. Comput. Assist. Radiol. Surg..

[11] Beigi P., Rohling R., Salcudean T., Lessoway V.A., Ng G.C. (2017). Detection of an invisible needle in ultrasound using a probabilistic SVM and time-domain features. Ultrasonics.

[12] Mwikirize C., Kimbowa A.B., Imanirakiza S., Katumba A., Nosher J.L., Hacihaliloglu I. (2021). Time-aware deep neural networks for needle tip localization in 2D ultrasound. Int. J. Comput. Assist. Radiol. Surg..

[13] Amin Z.T.A., Maryam A.J., Hossein M., Mirbagheri A., Ahmadian A. (2023). Spatiotemporal analysis of speckle dynamics to track invisible needle in ultrasound sequences using convolutional neural networks: A phantom study. Int. J. Comput. Assist. Radiol. Surg..

[14] Yan W., Ding Q., Chen J., Yan K., Tang R.S.-Y., Cheng S.S. (2023). Learning-based needle tip tracking in 2D ultrasound by fusing visual tracking and motion prediction. Med. Image Anal..

[15] Wang R., Tan G., Liu X. (2024). TipDet: A multi-keyframe motion-aware framework for tip detection during ultrasound-guided interventions. Comput. Methods Programs Biomed..

[16] Khosravi B., Li F., Dapamede T., Rouzrokha P., Gamblea C.U., Trivedic H.M., Wylesb C.C., Sellergrend A.B., Purkayasthae S., Ericksona B.J. (2024). Synthetically enhanced: Unveiling synthetic data’s potential in medical imaging research. EBioMedicine.

[17] Wang J., Wang K., Yu Y., Lu Y., Xiao W., Sun Z., Liu F., Zou Z., Gao Y., Yang L. (2025). Self-improving generative foundation model for synthetic medical image generation and clinical applications. Nat. Med..

[18] Xu J., Hua Q., Jia X., Zheng Y., Hu Q., Bai B., Miao J., Zhu L., Zhang M., Tao R. (2024). Synthetic breast ultrasound images: A study to overcome medical data sharing barriers. Research.

[19] Croitoru F.A., Hondru V., Ionescu R.T., Shah M. (2023). Diffusion models in vision: A survey. IEEE Trans. Pattern Anal. Mach. Intell..

[20] Ho J., Jain A., Abbeel P. Denoising diffusion probabilistic models. Proceedings of the Advances in Neural Information Processing Systems (NIPS).

[21] Ronneberger O., Fischer P., Brox T. U-net: Convolutional networks for biomedical image segmentation. Proceedings of the Medical Image Computing and Computer-Assisted Intervention (MICCAI).

[22] Prez P., Gangnet M., Blake A. (2003). Poisson image editing. ACM Trans. Graph..

[23] Ge Z., Liu S., Wang F., Li Z., Sun J. (2021). Yolox: Exceeding yolo series in 2021. arXiv.

[24] Lin T.Y., Maire M., Belongie S., Hays J., Perona P., Ramanan D., Dollár P., Zitnick C.L. (2014). Microsoft coco: Common objects in context. Proceedings of the Computer Vision–ECCV 2014: 13th European Conference.

[25] Song J., Meng C., Ermon S. (2020). Denoising diffusion implicit models. arXiv.

[26] Jiang H., Imran M., Zhang T., Zhou Y., Liang M., Gong K., Shao W. (2025). Fast-DDPM: Fast denoising diffusion probabilistic models for medical image-to-image generation. IEEE J. Biomed. Health Inform..

[27] Chen Y., Cao Y., Hu H., Wang L. Memory enhanced global-local aggregation for video object detection. Proceedings of the IEEE/CVF Conference on Computer Vision and Pattern Recognition (CVPR).

[28] Shi Y., Wang N., Guo X. YOLOV: Making still image object detectors great at video object detection. Proceedings of the AAAI Conference on Artificial Intelligence.

[29] Wang H., Tang J., Liu X., Guan S., Xie R., Song L. Ptseformer: Progressive temporal-spatial enhanced transformer towards video object detection. Proceedings of the European Conference on Computer Vision (ECCV).

[30] Li J., Yu Z., Du Z., Zhu L., Shen H.T. (2024). A comprehensive survey on source-free domain adaptation. IEEE Trans. Pattern Anal. Mach. Intell..

[31] Fogante M., Carboni N., Argalia G. (2022). Clinical application of ultra-high frequency ultrasound: Discovering a new imaging frontier. J. Clin. Ultrasound.

